# Views of Specialist Clinicians and People With Multiple Sclerosis on Upper Limb Impairment and the Potential Role of Virtual Reality in the Rehabilitation of the Upper Limb in Multiple Sclerosis: Focus Group Study

**DOI:** 10.2196/51508

**Published:** 2024-04-26

**Authors:** Amy Webster, Matthieu Poyade, Elaine Coulter, Lisa Forrest, Lorna Paul

**Affiliations:** 1 School of Health and Life Sciences Glasgow Caledonian University Glasgow United Kingdom; 2 School of Simulation and Visualisation Glasgow School of Art Glasgow United Kingdom

**Keywords:** virtual reality, multiple sclerosis, upper limb rehabilitation, coproduction, activities of daily living, exercise games, upper limb impairment

## Abstract

**Background:**

Finding enjoyable and effective long-term approaches to rehabilitation for improving the upper limb (UL) function of people with multiple sclerosis (MS) is challenging. Using virtual reality (VR) could be a solution to this challenge; however, there is a lack of reporting on the views of people with MS and clinicians on VR-based approaches and recommendations for games for rehabilitation.

**Objective:**

This study aims to identify common UL problems and their related current therapeutic approaches for people with MS, and to explore the opinions of people with MS and specialist clinicians on VR and obtain suggestions for the development and design of VR games.

**Methods:**

Separate focus groups were conducted with people with MS, recruited through the MS Society UK’s research network, and clinicians, recruited through the MS Trust Therapists in MS network. A total of 10 people with MS (2 focus groups) and 8 clinicians (5 physiotherapists, 2 occupational therapists, and 1 MS nurse in 2 focus groups) were involved. The focus groups were recorded and transcriptions were analyzed using theme-based content analysis.

**Results:**

People with MS commonly reported that their UL problems interfered with activities of daily living and resulted in the loss of meaningful hobbies such as writing. Many people with MS neglected UL exercise and found strategies for adapting to the UL impairments. Similarly, clinicians stated UL rehabilitation was neglected within their service and that it was challenging to find interesting treatment strategies. VR was suggested by both participant groups as a solution, as it was convenient for people with MS to access and it could provide a more engaging and disguised approach to exercise. There were shared concerns with cybersickness and disengagement with using VR approaches. Both groups agreed games should be meaningful and adaptable for users but suggested different VR activities, with clinicians suggesting games directly reflecting activities of daily living and people with MS suggesting more abstract activities.

**Conclusions:**

VR was well received by both people with MS and clinicians for UL rehabilitation. Recommendations were made for the development of VR rehabilitation games which are personalized and customizable for the varying abilities of people with MS.

## Introduction

### Background

Multiple sclerosis (MS) is an inflammatory demyelination disorder of the central nervous system that is estimated to affect 2.8 million people worldwide [1]. Over a third of the people with MS have upper limb (UL) dysfunction, including weakness, tremors, and spasms in one or both ULs [2]. This can result in difficulties with activities of daily living (ADL), negatively impacting quality of life and the likelihood of remaining in employment [3,4]. Problems specifically with dexterity are related to higher health care costs [5] and a higher association with depression-like psychological measures compared to problems with lower limb function [6]. Rehabilitation and physical exercise improve motor function for people with MS [7,8]. The evidence regarding UL rehabilitation is lacking in comparison with the lower limb, despite the high frequency of UL impairments and their impact on ADL [9]. In addition, there are particular challenges in finding effective yet motivating rehabilitation strategies in MS due to the long-term, progressive nature of the disease and diversity of symptoms [10].

Virtual reality (VR) is increasing in popularity in rehabilitation research and is proposed as a possible approach to encourage long-term rehabilitation [11]. VR includes digital environments that often simulate real-world experiences with reported benefits of high motivation and engagement, with real-time feedback [12]. VR has shown promising results within MS populations, but this evidence is limited in comparison with stroke, especially regarding UL function [13]. Our systematic review, investigating the effect of VR in improving UL function in MS, found early, but limited, evidence suggesting VR has the potential to improve function in people with MS [14]. There was also a low number of dropouts in most studies within the review, supporting that VR could improve adherence compared with conventional rehabilitation; therefore, VR could be useful in conditions such as MS, where prolonged rehabilitation is required.

VR is often investigated alongside video games played within a VR setting, which can be commercially available or specifically tailored games designed with a target population in mind. Commercially available exercise games, targeted at a healthy population, can be unsuitable for disabled individuals and lead to discouragement and anxiety [15]. It is beneficial to involve a sample of target users in the creation and development of effective VR-based gamified approaches [16]. This process is known as coproduction [17]. To date, no study has systematically coproduced VR games specifically for UL rehabilitation in people with MS.

### Objectives

The aims of this study were to determine the views of people with MS and specialist clinicians on UL dysfunction or function in MS, challenges faced by clinicians when delivering UL therapy, barriers and motivators for exercise in MS, opinions on VR, and suggestions for development and design of VR games. These findings will guide the future development of VR applications and interventions for UL rehabilitation for people with MS.

## Methods

### Ethical Considerations

Ethics approval for this study was provided by the School of Health and Life Sciences Ethics Committee at Glasgow Caledonian University (HLS/PSWAHS/20/002). Informed consent was obtained from the participants and clinicians.

### Recruitment

The study aimed to recruit up to 12 people with MS and 12 specialist MS clinicians to take part in online focus groups. The sample size was determined in line with the design of other similar studies and general recommendations for qualitative analysis [18,19]. To be included in the study, people with MS were required to be aged ≥18 years and have a diagnosis of MS (self-reported) with self-reported UL impairment. Clinicians were required to have experience (any duration) in delivering MS rehabilitation within the National Health Service (NHS) or the third sector. In addition, all participants were required to have access to and the ability to operate videoconference software. There were no specified exclusion criteria. Participants with MS were identified through the MS Society UK’s research network, which advertised the study to its members. Those who were interested in participating contacted the research team directly and were emailed a participant information sheet. In terms of recruitment of clinicians, the MS Trust Therapists in MS network advertised the study to its members. Interested clinicians contacted the research team and were emailed a participant information sheet.

### Coproduction Focus Groups

**Figure 1:**
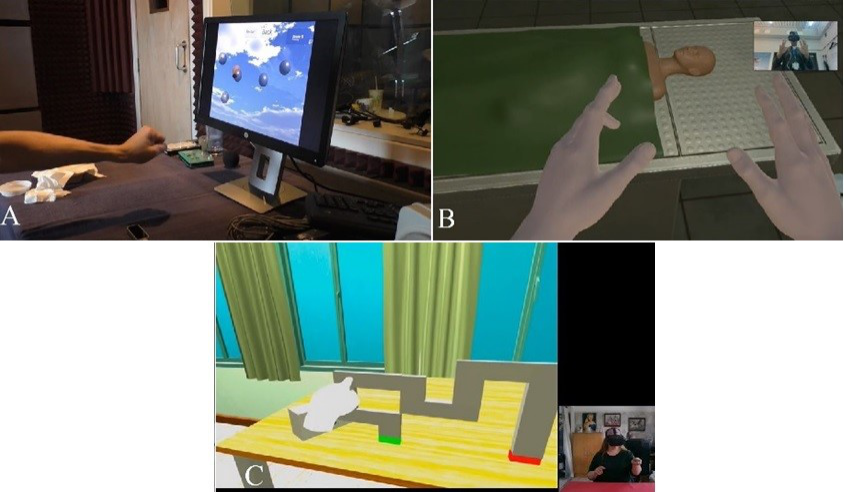
Stills from videos shared with participants during focus groups, demonstrating different virtual reality technology. (A) Video 1 shows Leap Motion only; (B) video 2 shows Leap Motion and Oculus Rift; and (C) video 3 shows Oculus Quest.

The focus groups for people with MS and clinicians were conducted separately with a maximum of 5 people per focus group. To comply with COVID-19 pandemic regulations at the time, focus groups were held online using Zoom (Zoom Video Communications) or Teams (Microsoft Corp) videoconference software; this also provided an opportunity for recruitment of participants from across the United Kingdom and Ireland. The focus groups were conducted in a semistructured style using a focus group schedule split broadly into three sections important for the development of VR interventions for UL problems in people with MS: (1) UL dysfunction and exercise or therapy; (2) opinions on VR; and (3) suggestions for development and design of any developed VR games (Multimedia Appendix 1). In addition, clinicians were asked what information and feedback they would want from a patient’s VR therapy session. The questions included prompts that allowed more targeted responses from participants regarding their experiences and views [20]. Within the focus groups, participants were shown three videos demonstrating different commercially available head-mounted devices (HMDs) and hand-tracking devices: (1) a nonimmersive VR set up using a Leap Motion controller and computer monitor, which is a hand motion capture device that allows users to visualize their hand movements and interact with virtual environments; (2) immersive VR using the Oculus Rift HMD with a mounted Leap Motion device for hand tracking; and (3) immersive VR using the Oculus Quest, with in-built hand tracking (Figure 1 [21,22]; Multimedia Appendix 2). Videos were shown as participants were unable to try these devices since the focus groups were online due to the COVID-19 pandemic. These videos attempted to contextualize and demonstrate the different VR and motion capture devices in terms of users interacting with environments, possible hand movements, and previous games developed from prior research. After watching the videos, participants were encouraged to share their initial thoughts on each of the technologies. The focus groups involving people with MS and clinicians lasted approximately 90 minutes and 60 minutes, respectively. The focus groups were facilitated by a female researcher (AW) who had been involved in the recruitment of participants and an additional senior, female researcher (LP) attended.

### Data Analysis

All focus groups were audio recorded and transcribed verbatim. Qualitative analysis of the data was performed based on theme-based content analysis (TBCA) as described in the study by Neale and Nichols [23]. This qualitative method groups responses into content-related themes to enable researchers to view the user preferences more easily and has been used to influence the development or evaluation of a VR environment [23-25]. TBCA is a flexible qualitative data analysis method that involves five key steps: (1) data collection, (2) data collation, (3) raw theme definition and classification, (4) higher order theme selection, and (5) presentation of classification matrix [23]. Owing to the large number of higher order themes, we added an additional step by grouping the higher order themes into main themes. The raw themes were assigned independently by 2 researchers in the transcripts of people with MS (AW and LF) and clinicians (AW and LP). After agreement on the raw themes, the responses were then independently grouped by 2 researchers (AW and LP) into higher order themes. Any discrepancies in assigning the themes were resolved through consultation with a third reviewer, if necessary. Once the higher order themes were determined, the main themes were determined by 2 researchers (AW and LP). The main themes with their associated raw and higher order themes are presented in tables. The raw and higher order themes were quantified manually within the matrix based on the number of responses necessary to display popularity or consensus [23], and example quotes for each higher order theme were included. Focus groups of people with MS and clinicians were analyzed separately to allow comparison of the findings between the 2 groups.

## Results

### Participant Demographics

A total of 10 people with MS were recruited to the study and took part in 1 of 2 focus groups, each of which had 5 participants. Most participants with MS were female (7/10, 70%), with a mean age of 56.4 (SD 16.5) years and a mean time since diagnosis of 14.4 (SD 12.3) years. Participants had varying MS types (Table 1).

A total of 8 clinicians were recruited (5 physiotherapists, 2 occupational therapists, and 1 MS specialist nurse). Among them, 6 participants worked in the NHS and 2 worked in other settings. There were 2 focus groups for clinicians with 4 participants in each group. All clinicians were female, with a mean age of 46.2 (SD 9.6) years, and the mean length of experience was 17.9 (SD 10. 2) years.

**Table 1:** Demographic details of people with multiple sclerosis.

Participant ID	Age (years; mean 56.4, SD 16.5)	Sex	Multiple sclerosis type	Time since diagnosis (years; mean 14.4, SD 12.3)
P1	60	Female	SPMS^a^	30
P2	38	Female	RRMS^b^	4
P3	68	Male	SPMS	35
P4	58	Female	SPMS	1
P5	42	Female	SPMS	11
P6	28	Female	RRMS	3
P7	56	Female	PPMS^c^	5
P8	70	Male	PPMS	16
P9	60	Male	SPMS	12
P10	84	Female	SPMS	27

^a^SPMS: secondary progressive multiple sclerosis.

^b^RRMS: relapsing-remitting multiple sclerosis.

^c^PPMS: primary progressive multiple sclerosis.

### People With MS: TBCA

#### Overview

Following TBCA of the focus groups of people with MS, 20 higher order themes were determined based on the grouping of the assigned raw themes. These 20 higher order themes were grouped into four main themes: (1) Impact of MS on the UL; (2) Exercising with MS; (3) Views of people with MS on VR; and (4) Recommendations for development and user requirements (Table 2). A full version of this table, including more example quotes from participants, is available in Multimedia Appendix 3.

**Table 2:** Main, higher order, and raw themes from theme-based content analysis of people with multiple sclerosis focus groups.

Main theme and higher order themes (number of responses)			Raw themes (number of responses)
**Impact of MS^a^ on the UL^b^**			
	Interference with functional activities (35)	Dressing (8); eating (6); dropping items (5); writing (5); grooming (3); dependence on others for activities of daily living (3); carrying items (3); and traveling (2)	
	Symptoms and signs that impact activities (25)	Fatigue (10); numbness (6); sensory overload (4); weakness (3); tremors (2); proprioception (2); and coordination (1)	
	Strategies people with MS adopt to assist with ADL^c^ (24)	Strategies for functional activities (8); adapting (7); making a difference (5); technology assistance (2); and mobility-assistance equipment (2)	
	Struggle with loss of meaningful activities and skills (14)	Loss of skills (6); impact of losing ability to write (4); and keeping meaningful activities (4)	
	UL actions people with MS find difficult (13)	Dexterity (6); range of motion (4); and grip (3)	
	Sharing and sympathy (13)	Sharing strategies (4); sharing advice on exercise (4); taking advice (3); and sympathizing (2)	
	Difficulty with progression and unpredictable nature of MS (10)	Variation in MS (6); unpredictable (2); and progression (2)	
**Exercising with MS**			
	Views and attitudes on exercise (49)	Maintenance (10); negative perceptions of exercise (8); keeping muscle strength (8); determined to exercise (7); benefits of exercise (6); multitask approach (4); legs focus (3); and in control (3)	
	Previous experience of UL rehabilitation or exercise (40)	Outcomes from UL exercise or rehabilitation (12); neglecting UL exercise or rehabilitation (10); UL equipment (6); UL physiotherapy (4); driven for UL exercise (3); UL exercise resources (3); and adherence (2)	
	Barriers to exercise (28)	Personal barriers (8); environmental barriers (8); COVID-19 barriers (7); and verbal disengagement (5)	
	Facilitators to exercise (28)	Verbal encouragement (10); health care professionals (8); MS center (4); gym facilitators (3); and pushing self for results (3)	
	Adverse effects of exercise (11)	Induce symptoms (4); tiring (3); recovery time after exercise (2); affecting socializing (1); and overdoing exercise (1)	
	Approaches to exercise used by people with MS (26)	Routine (7); exercise bikes (6); exercise aims (5); low impact or stretching exercise (4); and physiotherapy approaches (4)	
	Views on group vs individual exercise (26)	Competition in exercise (10); motivation of group exercise (5); downsides of group exercise (5); importance of socializing in exercise (2); camaraderie (2); enjoyment (1); and interest in group exercise (1)	
**Views of people with MS on VR^d^**			
	Positive views on VR (55)	Home use (9); outcome benefits (6); personal opinions on VR (5); fun (5); adaptable (5); positives of technology (5); wireless convenience (5); accessibility convenience (4); incentives (3); meaningful (3); online socializing (2); and immersion (1)	
	Negative views on VR (40)	Cybersickness (17); HMD^e^ discomfort (6); technology discomfort (5); HMD dislike (3); disengagement (3); accessibility concerns (3); and unsuitability (3)	
	Views on trying or participating in VR rehabilitation (25)	Openness to VR (12); challenging (4); safety considerations (3); need results (2); technology considerations (2); and unsuitable for them (2)	
**Recommendations of people with MS for development and user requirements**			
	Considerations for development of VR games (84)	Mindful of target audience (9); tracking progress (8); discouragement of feedback (8); knowing UL outcomes (7); end result (6); score targets (6); challenging self (6); competition in games (5); education (5); time feedback (4); supervision (4); community involvement (3); multipurpose (3); continuous development (3); be fun (3); hardware (2); and learning patterns concern (2)	
	Suggestions for VR activities (36)	Suggested UL actions (9); game ideas (7); real-life vs abstract tasks (4); haptic activities (4); strength in games (4); writing and drawing (3); demonstrated games (3); additional objectives (2); and atmosphere (1)	
	Importance of choice (23)	Offer different movements (8); having a variety of games (6); personal preferences (6); and variety of different levels (3)	

^a^MS: multiple sclerosis.

^b^UL: upper limb.

^c^ADL: activities of daily living.

^d^VR: virtual reality.

^e^HMD: head-mounted device.

#### Impact of MS on the UL

The most common higher order theme was “Interference with functional activities” with 35 responses (Table 2). Participants reported a wide range of activities they found difficult to perform due to their MS, the most frequent being ADL, including personal care, eating, and carrying heavy items. “Symptoms and signs that impact activities” had the second highest number of responses (n=25), where participants particularly noted the impact of fatigue on activity (n=10); however, sensory problems such as numbness and pins and needles were also highlighted. Other MS symptoms impacting UL function were, for example, weakness, tremors, and coordination problems. In “Strategies people with MS adopt to assist with ADL” (n=24), because of losing function, participants discussed the use of assistive equipment, for example, button fasteners, specialized cups, and voice control. Other strategies included using their less affected hand or pacing to manage fatigue. The remaining 4 higher order themes had fewer responses. In brief, dexterity, range of joint movement, and grip were the main “UL actions people with MS find difficult” (n=13). These were often compounded by the unpredictability and progressive nature of MS (“Difficulty with progression and unpredictable nature of MS,” n=10). Participants reported the emotional impact of losing the ability to carry out personal and meaningful activities specifically because of loss of UL function (“Struggle with loss of meaningful activities and skills,” n=14), with one participant stating the following:

I used to be a writer and it was very, very hard because I couldn’t write anymore...I was really motivated [to relearn writing], felt really cut off from the world.

P8; age 70 years; male participant with primary progressive multiple sclerosis

The final higher order theme was “Sharing and sympathy” (n=13 responses), where participants empathized and shared experiences and suggestions of assistive equipment.

#### Exercising With MS

Most responses under this main theme related to “Views and attitudes on exercise” (n=49; Table 2). Participants were motivated to exercise with a "*use it or lose it*” attitude and a desire to, if not improve then at least maintain, their function and prevent further deterioration. Participants also described negative perceptions of exercise, such as finding it “*very boring*” and guilt from not participating in exercise. In “Previous experience with UL rehabilitation or exercise” (n=40), many participants (5/10, 50%) discussed not undertaking any UL exercise or rehabilitation, currently or previously. Many UL programs previously undertaken by some participants aimed to build strength, reduce pain, and improve hand function with varying outcomes. There were similar numbers of responses in terms of “Barriers to exercise” (n=28) and “Facilitators to exercise” (n=28). Personal barriers to exercise included comorbidities, MS symptoms (fatigue, pain, and bladder and bowel dysfunction), difficulty using exercise equipment, and expense. The COVID-19 pandemic had negatively impacted the participants’ exercise due to services closing down. Environmental barriers to exercise included lack of local facilities and not having space to exercise at home. Verbal encouragement was described as both a barrier (could be off putting) and a facilitator (motivating) to exercise. Other facilitators were seeing improvements, feeling motivated, and the attitudes of health care professionals, personal trainers, and carers. Conversely, health care professionals with a lack of experience in MS overwork people with MS, leading to exhaustion (“Adverse effects of exercise,” n=11). Participants undertook many different forms of exercise (“Approaches to exercise used by people with MS,” n=26), including exercise bikes, Pilates and yoga, dog walking, and gym exercises. There were varying “Views on group versus individual exercise” (n=26). Some found competition within a group to be motivating while others did not, with one participant suggesting social support and camaraderie was more important than competition:

I’m not too fussed about being in competition with others, but if it was a more social thing that would maybe encourage me to perhaps join in a group that’s doing something together.

P4; age 58 years; female participant with secondary progressive multiple sclerosis (SPMS)

Negative aspects of group exercise included the fear of letting others down.

#### Views on VR

The initial reaction to VR was positive (“Positive views on VR,” n=55; Table 2). Participants stated it looked fun or enjoyable with the potential to improve or maintain muscle strength, dexterity, and spatial awareness, especially with repeating the actions and concurrently perhaps learning a new skill (for example, playing the piano):

I think [VR’s] still very good because... it’s...maintaining those motor skills that is so easily slip away when you’re not using them.

P9; age 60 years; male participant with SPMS

There were positive comments in relation to the convenience and accessibility of VR facilitating exercise at home at a suitable time and eliminating travel to physiotherapy services and gyms. Participants highlighted that the wireless HMD was more convenient as it was portable and did not need a computer. The advantage of linking up with others online was raised. However, “Negative views on VR” (n=40) were related to concerns regarding cybersickness, linked to dizziness and balance problems:

With MS a lot of people suffer from nausea or motion sickness. That can be a concern for the headsets.

P6; age 28 years; female participant with relapse and remitting multiple sclerosis

Other negative responses related to the HMD discomfort regarded weight, usability concerns, wearing it with glasses, and being disconnected from the real world. Two participants indicated that interest in VR may reduce over time. Participants were also concerned about fatigue and the usefulness of VR for UL sensory dysfunction. Most participants (6/10, 60%) expressed they were open to trying VR (“Views on trying or participating in VR rehabilitation,” n=25), but would like to understand the benefits, long-term outcomes, and any safety issues.

#### Recommendations for Development and User Requirements

With regard to “Considerations for development of VR games” (n=84), a variety of UL movements was desirable with clarity in terms of the aim and outcome in relation to the UL being important (Table 2). Competition within the VR games, interacting with others or challenging themselves, were frequently discussed as being motivating. Tracking improvements during VR gameplay was vital to some participants, including monitoring improvements in score, exercise time (rather than countdown which could be stressful), and progressive challenges. The games should offer the ability to challenge users, with one participant saying the following:

That challenge to try and be better the next time, whereas if you’ve got no idea...you’ve got nothing to fight against or to work against.

P10; age 84 years; female participant with SPMS

Conversely, other participants emphasized the potential demotivating effect of feedback given the progressive nature of MS, by warning that score feedback should not be “*disheartening*,” and should therefore be made optional to the user. There was a strong feeling that the VR games should be “*fun*” with abstract gameplay potentially being more fun. Participants felt that demonstrations and supervision to assess progress were important. They also stated that the VR games had to account for the differences in the ability of people with MS and that older people may need more basic VR games. The idea of the VR games having an educational outcome or in learning a new skill was suggested to help with engagement. Participants suggested that reaching, punching, and other aerobic activities could be incorporated (“Suggestions for VR activities,” n=36). Having haptic approaches was frequently proposed with gripping, squishing games, such as kneading bread. Participants proposed activities with a cognitive element, such as a puzzle or maze, and whole limb movements, such as Whack-a-Mole (Mattel), writing or drawing. Participants liked the VR piano which had been demonstrated. There was a variety of opinions in terms of abstract or real-life activity with most preferring abstract games but some ADL-type activity was also suggested. “Importance of choice” (n=23) related to having variety in games, UL movements, and levels of difficulty with abstract games or real-life gamified tasks, with 1 participant declaring the following:

I’d like to make sure I’m not doing a whole lot of exercises that are all doing the same things...Got to be mixing them up: one for coordination, one for dexterity.

P1; age 60 years; female participant with SPMS

### Clinicians: TBCA

#### Overview

From the clinician focus groups, there were 15 higher order themes grouped into four main themes: (1) Current methods and challenges for delivering UL rehabilitation; (2) Clinicians’ views on VR; (3) Recommendations for development and user requirements; and (4) Implementation of VR into practice (Table 3). A full version of this table, including more example quotes from participants, is available in Multimedia Appendix 4.

**Table 3:** Main, higher order, and raw themes from theme-based content analysis of clinician focus groups.

Main themes and higher order themes (number of responses)			Raw themes (number of responses)
**Current methods and challenges for delivering UL^a^ rehabilitation**			
	Challenges clinicians face when delivering exercise for people with MS^b^ (52)	MS-specific challenges (13); patient adherence (11); service challenges (9); UL-related challenges (7); patient differences (6); challenges with current methods of delivery (4); COVID-19 impacts (2)	
	Recommended UL exercises for people with MS (29)	Actions (10); systematic approach (7); functional tasks (6); strength and range of movement (5); relapse care (1)	
	Experience with long-term, progressive condition (24)	Deterioration (11); acceptance in patients (8); difficulty with patient improvements (5)	
	Factors clinicians consider when prescribing exercise for the UL (22)	Meaningful and patient-focused (9); patient assessments (6); symptoms (4); repetition (3)	
	Current methods of UL exercise delivery for people with MS (15)	Technological approaches (4); programs (4); accessible equipment (3); clinician routines (2); patient lead (2)	
	Socializing in exercise (14)	social motivation (6); support (5); recommending social exercise (3)	
**Clinicians’ views on VR^c^**			
	Positive views on VR (50)	Solutions to current challenges (10); personal opinions on VR (7); facilitating movements or tasks (6); VR-specific qualities (6); meaningful (5); engagement (5); visualization (4); novel (3); cognitive appeal (2); adaptability (2)	
	Negative views on VR (38)	Disengagement (10); cybersickness and safety (8); HMD^d^ discomfort (7); accessibility concerns (5); feedback concerns (5); validity concerns (3)	
	Questioning benefits and the unknowns of VR (14)	Questioning purpose of VR (4); questioning benefits of VR (4); neural mechanisms (3); research (2); different VR systems (1)	
**Clinicians’ recommendations for development and user requirements**			
	Considerations for developing VR games for people with MS (41)	Communication between clinician and patient (12); purposeful (7); social components (7); selecting tasks (4); slower tasks (3); competition (3); feedback for clinician (3); positive feedback (2); end point (2)	
	Suggestions for VR activities (18)	Activities of daily living (6); hobbies (6); objectives (6)	
	Importance of choice (15)	Preferences (6); having variety (5); setup (4)	
**Implementation of VR into practice**			
	Suggestions for incorporation of VR into practice (18)	Home use (7); VR in clinics (7); long-term treatment (4);	
	Challenges with implementation of VR into practice (24)	Funding (7); demanding on services (6); availability of equipment (5); risk (3); adjustment (2); uncertainty of practice (2)	
	Finding the target audience for VR (8)	Who would use VR (3); niche group (3); age (2)	

^a^UL: upper limb.

^b^MS: multiple sclerosis.

^c^VR: virtual reality.

^d^HMD: head-mounted device.

#### Current Methods and Challenges for Delivering UL Rehabilitation

“Recommended UL exercises for people with MS” (n=29) included strength training and active movements related to functional activity, such as hand-to-mouth movements (Table 3). Treatment for the UL often involved equipment such as Therabands and Theraputty but also technology such as the Gloreha robotic system and functional electrical stimulators with different models of care for UL exercises described as part of community-based classes, within third sector organizations and online programs (“Current methods of UL exercise delivery for people with MS,” n=15). Within “Factors clinicians consider when prescribing exercise for the UL” (n=22), most responses were regarding meaningful and goal-focused exercises. Clinicians also considered the patient’s symptoms, for example, spasticity, pain, and the ability of patients. The importance of repetition of movement was reinforced. Most responses (n=52) were in relation to “Challenges clinicians face when delivering exercise for people with MS.” Clinicians expressed that UL-focused exercise was neglected compared to the lower limb and the challenge of making UL exercise interesting:

A bit more difficult for upper limb things...it’s much easier to maybe...go for a walk with somebody or you know, or cycle or whatever. Upper limb is maybe a wee bit more difficult.

C6; physiotherapist

Clinicians also mentioned the use of Theraputty described as “*juvenile*” and lists of exercises “*boring*.”

Service-related challenges included limited time and capacity to see patients and large geographic areas to cover. Other challenges were keeping patients engaged with exercise in the long term, especially at home, and finding an activity that would be attractive to patients. Under “Experience with long-term, progressive condition” (n=24), clinicians raised being realistic about improvements with a progressive condition while also keeping patients motivated, minimizing deterioration or maintenance, rather than improving:

Trying to motivate people with progressive MS, you’re trying to get them to continue to maintain where they are rather than improve.

C5; occupational therapist

Clinicians expressed the positive benefits of “Socializing in exercise” (n=14) for support and motivation.

#### Clinicians’ Opinions on VR

Clinicians were very positive about VR (n=50), describing it as being interactive, fun, meaningful, and a novel potential approach to rehabilitation, which could help engagement (Table 3). They were positive about the escapism aspect and the potential to improve mental health:

What appeals about VR stuff is that it is focused and takes you into a different place...You’re doing tai chi on a beautiful, Japanese garden rather than actually in your grumpy living room...I think even that in terms of the escapism aspect, maybe from a mental wellbeing.

C1; physiotherapist

Clinicians liked the visual feedback to help with, for example, coordination, but which could also reinforce movements and introduce a cognitive component. Clinicians commented that VR provided the opportunity to undertake activities not possible within the clinic and to exercise without the activity seeming like an exercise. Most of the “Negative views of VR” (n=38) were regarding patient safety using VR headsets, especially cybersickness, including dizziness and disorientation, specifically in patients with vestibular issues. Other general concerns with HMDs were usability with glasses, the weight of the HMD, and feeling claustrophobic. Clinicians suggested that VR activities should not be too simplistic to avoid patronizing patients and at an appropriate skill level. The longevity of engagement of patients after the initial novelty was questioned. Clinicians also questioned the use of VR for activities that can be done in the real world and similarly how VR activities might translate to real function. The importance of feedback on the quality of movement as well as the quantity was highlighted. Finally, accessibility and digital poverty were also raised. The final higher order theme was “Questioning benefits and the unknowns of VR” (n=14), where some clinicians felt there was insufficient evidence on the purpose and benefits of VR and its effect on neural mechanisms:

I think it’s important to think about how is [VR] different to just doing [activities] in real life as well...What can you augment in your rehab through this virtual reality that you can’t just do in real life anyway?

C7; physiotherapist

#### Recommendations for Development and User Requirements

Under “Considerations for developing VR games for people with MS” (n=41), clinicians discussed the importance of the VR games having purposeful activity, translation of tasks into real life, and having an end point (Table 3). The games should consider movements of individual joints of the UL with extension movements at the wrist and fingers being important as where people with MS lose the most function. Games should incorporate strength, coordination, proprioception, and range of motion exercise as well as exercises for the core. Feedback was important, with clinicians able to monitor the program. Clinicians were not interested in scores for the games but wished feedback on the quality of the movements and patient engagement. Clinicians stated that undertaking VR activities with others or in group settings with elements of competition was desirable. Clinicians provided “Suggestions for VR activities” (n=18), including ADL such as putting on makeup; writing or chopping vegetables; and hobbies including pottery, sewing, or piano playing. Clinicians raised the “Importance of choice” (n=15) in the VR setup, choice of games, and choice within games, for example, levels of difficulty, to appeal to as many people as possible:

I think, it is about having a variety of things that push as many buttons with patients that you can manage and cover as many options as you can.

C2; physiotherapist

#### Implementation of VR Into Practice

Under “Suggestions for incorporation of VR into practice” (n=18), clinicians felt long-term, regular use of VR was needed for positive outcomes (Table 3). Home use was felt to encourage frequent use, with clinicians monitoring progress remotely, thus saving in person contact time. There were a number of “Challenges with implementation of VR into practice” (n=24) with cost and funding (service and individual) being the most commonly reported, which included potential increased demand on services:

I know if I brought it to my bosses they would want a breakdown of cost of monthly rate, how are we going to utilise it, how often are we going to utilise it. What figures could we get from this particular item and what outcomes could we achieve.

C4; MS specialist nurse

Equipment-related challenges were ownership, availability, supply of equipment, and infection control. A full risk assessment would be required before implementation and guidance would be needed on intervention duration and frequency. Clinicians discussed for whom VR would be appropriate, in terms of age or other factors, and identified this as an area for future research (“Finding the target audience for VR,” n=8).

## Discussion

### Principal Findings

**Figure 2:**
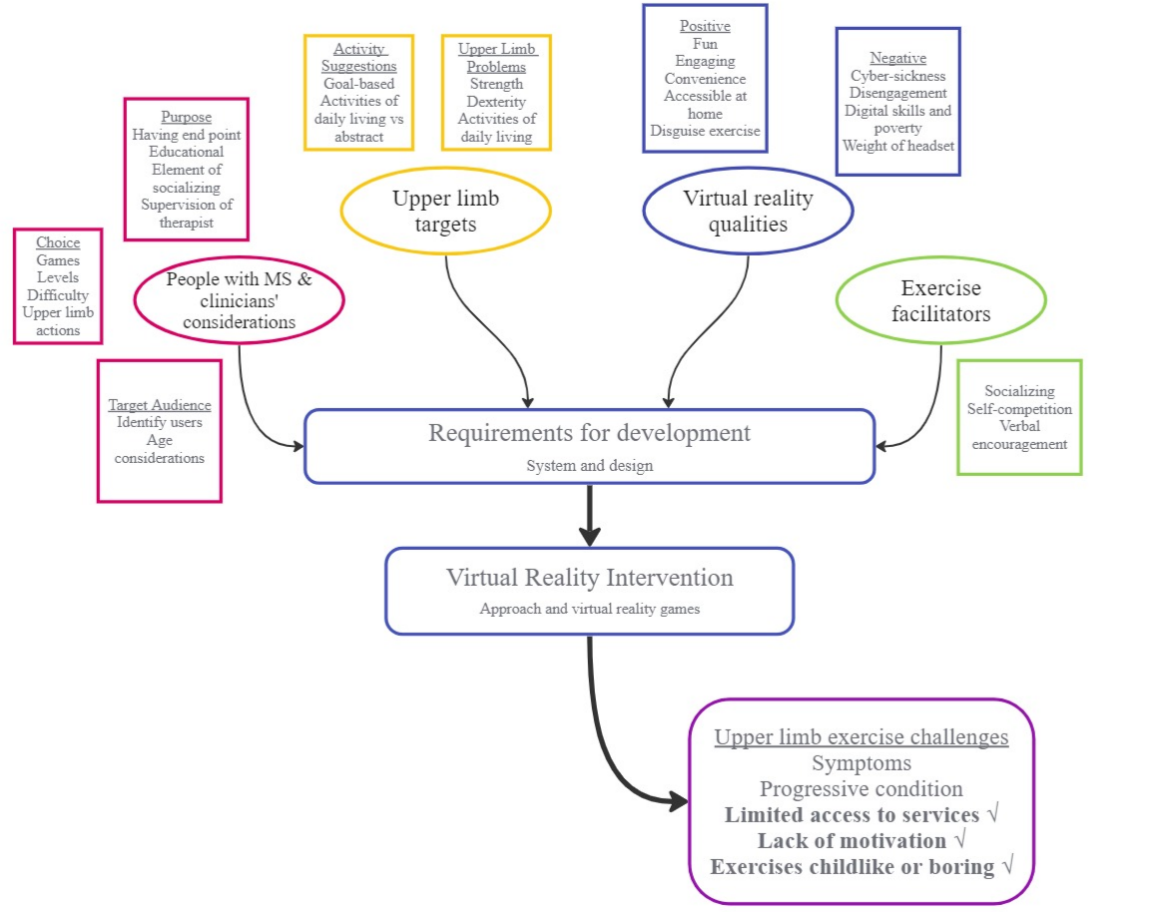
Flow diagram of the results from theme-based content analysis of people with multiple sclerosis (MS) and clinicians’ coproduction focus groups and how this will guide the requirements for developing virtual reality–based games or interventions, which will aim to tackle certain achievable upper limb exercise challenges within MS.

This study aimed to explore the views of people with MS and clinicians on UL impairment associated with MS and the potential role of VR as a rehabilitation approach to address this impairment. The discussion focuses on the combined findings from the 2 groups of participants: people with MS and clinicians (Figure 2). Figure 2 is a visual representation of the principal findings based on the higher number of responses assigned, which should inform the development of VR applications and interventions aiming to improve the UL function for people with MS and how VR could tackle challenges of existing UL exercise raised by clinicians and people with MS in this study. The findings agree with those of previous studies that people with MS commonly have UL impairments that impact function, including problems with dexterity and ADL, which leads to loss of meaningful activities [26-28]. Despite UL difficulties, UL exercise was neglected due to MS symptoms, such as fatigue, lack of motivation, and dislike of exercise, as well as the challenges clinicians faced regarding time constraints and finding appropriate therapies that were not childlike or boring. Lack of focus on UL rehabilitation has been reported previously in MS [9] and in other long-term neurological conditions such as stroke [29]. The progressive and unpredictable nature of MS was raised by both groups, and consequently, clinicians raised the importance of setting realistic expectations with therapy, sometimes focusing on maintenance of function rather than improvement.

Both groups (people with MS and clinicians) were optimistic about the use of VR and believed VR could be a solution to their exercise challenges. Positive comments, including avoiding traveling, being accessible, and being engaging or fun addressed the identified barriers for UL rehabilitation. This concurs with previous VR studies [30,31] and specifically in UL rehabilitation in MS, with a recent home-based, feasibility study using the Oculus Quest 2 VR headset in which participants described VR as fun, interesting, and innovative [32]. Participants in the study by Kamm et al [32] suggested adding difficulty levels and scoring to their exercises, competitive elements previously described to be motivating by people with MS using nonimmersive exercise games delivered through the Nintendo Wii [33]. In this study, both groups were especially positive regarding the immersive approach of the Oculus Quest. Participants thought the escapism properties and visualization of movements could potentially “disguise exercise,” which may occur with the “fun” element of VR reducing the perception of exertion during exercise [34], therefore encouraging more UL therapy.

Negative views about VR were also expressed, mainly the potential for cybersickness. Cybersickness is thought to be caused by conflict of stimuli, leading to nausea, disorientation, and pain in the eyes and head [35]. Women are more susceptible to cybersickness [36], which is relevant in MS, with a higher number of women affected. Although cybersickness with VR has been reported previously in people with MS [37], there are development strategies for reducing cybersickness, such as designing VR activity with less overall movement within the virtual environment. Cybersickness is, however, thought to reduce over time with exposure to VR [38]. There were unnecessary concerns raised for those wearing glasses as the HMD can accommodate glasses, but there were valid concerns about the weight of the HMD for some users. Disengagement was another concern both groups expressed, with limited data on long-term adherence to VR in MS rehabilitation. Exercise is a behavioral intervention, and long-term adherence to exercise can be supported by evidence-based behavior change techniques [39]. These behavior change techniques, such as goal setting, rewards, and feedback, can be incorporated into VR games or activity to support long-term engagement in UL exercise. While VR can be more engaging than other methods of exercise [40], frequent performance, feedback on progress, and adjusting levels of difficulty can maximize VR engagement for those with long-term neurological conditions [41]. Finally, clinicians had specific concerns regarding digital poverty, the technical ability of people with MS, and insufficient technical services to support VR.

Considerations for VR game development align with user-centered design principles for VR in motor rehabilitation in survivors of stroke, such as being fun, tracking progress, having an element of competition, challenging oneself, and providing feedback [42], and are not specific to any clinical population. Participants raised that VR development should be mindful of the different end users (people with MS) who may differ in ability and preferences. Clinicians suggested VR would appeal to younger individuals with MS, whereas people with MS felt older people with MS might need more basic gameplay. While there is some, albeit limited, evidence for lower usability scores for older VR users compared with younger users, there can be higher user enjoyment [43], and there is moderate evidence for good usability of VR in older populations [44]; therefore, this concern may be overly cautious.

Consideration of the end user links to the importance of choice when designing VR interventions, with a variety of games to appeal to as many as possible. Participants felt the games should include different movements, levels of immersion, level of difficulty, or feedback on performance. Accommodating individual preferences is a key element for the design of VR games for rehabilitation, as it increases user engagement [45]. However, our previous systematic review found that a choice of games was rarely included in VR interventions in MS [14].

There were differing views in terms of the type of feedback people wished from VR. Some people with MS wanted to track scores and visualize results, which is supported by reward theories for users during both entertainment and serious games [46]. Conversely, concerns were raised about feedback potentially being discouraging or demotivating, especially given the variable nature of MS. As an example, countdown timers provide slight pressure to motivate players to increase engagement [47]; however, in this study, people with MS felt they could be stressful. Feedback on the duration of exercise completed was appealing to people with MS, as reported previously [19]. As well as the quantity of VR exercise, clinicians also wished feedback on the quality of movement when performing the games. Rehabilitation often involves highly repetitive movements to stimulate neuroplasticity; however, stroke specialist therapists have also previously reported concerns that quality of movement in VR rehabilitation for UL maybe sacrificed for a good gaming outcome [18], although this has not been explored in people with MS. Both groups were interested in the reported outcomes of using VR approaches which, if positive, would increase engagement.

Clinicians and people with MS felt VR activity had to be related to the patient’s personalized and meaningful goals, which is known to increase motivation in physiotherapy settings [48]; however, this is often neglected in VR regimes [14]. Goals need to be adjusted over time in a progressive condition, such as MS, and to avoid disengagement as raised earlier. Participants with MS frequently stated that their goals were related to not only improvement but also the maintenance of ability and the prevention of further deterioration. In terms of suggestions for VR activities, the groups differed with clinicians suggesting ADL or hobby simulations and people with MS being more ambivalent, stressing activity to be fun with a variety of real-life and abstract VR games. Previous studies of VR have often involved ADL activities such as cooking or other kitchen activity [49,50]. Although VR can provide a safe environment to practice ADL for people with mobility issues [51] people with MS in this study were less interested in ADL, especially kitchen simulations. Both groups suggested an “end result,” such as creating a drawing, or learning a new skill would be positive and facilitate a feeling of accomplishment. There were also suggestions from people with MS to incorporate haptic activities, such as grabbing and gripping. However, the user is not able to receive tactile feedback when interacting with a virtual environment, and handheld controllers may need to be considered for some VR activities [52]. Another solution could be to incorporate pseudo haptics, the use of different stimuli such as visual or auditory stimuli, to mimic a variety of haptic properties in a virtual environment [53]. This is an emerging field that could be explored in VR for people with MS. Similarly, as many of the participants suggested finger-related exercises, it is important that VR systems use good hand-tracking motion capture devices to allow visualization of the movement of fingers and wrists within a VR setting.

Many people with MS were supportive of VR for home use, as being more convenient and accessible. However, there was recognition that users needed demonstration of the technology and a level of clinician supervision. Assessing quality of movements and monitoring of patient progress are reported challenges for VR home use [54]. A recent study with a small number of participants found VR to be feasible for home-based UL rehabilitation in people with MS, after 3 supervised sessions [32], but larger studies of home-based VR for UL rehabilitation are required. There was agreement in both groups that an element of social interaction could be considered in the development of VR games. Generally, there is a lack of evidence on the effect of socialization within UL therapy, but it may improve adherence and motivation [55] and provide better outcomes [56]. Specifically in relation to VR, there is some evidence that social aspects increase motivation through competition [57], but participants in our study were more interested in self-competition rather than competing against others. This is similar to a study of a walking app for MS where users were less interested in sharing their goals or achievements with others [58].

### Strengths and Limitations

Recruiting participants through online sources may result in a biased sample, as those comfortable with technology and access to online services are more likely to take part. Being online allowed the involvement of people with MS with varying abilities and clinicians who worked in the NHS and the third sector across the United Kingdom. However, the online nature meant it was not possible for participants to physically test the VR equipment and explore their reactions. While it can also be challenging to engage all participants in online focus groups, this was resolved by asking questions using participants’ names or by getting participants to use the raise hand function within the videoconference software and encourage discussion between participants.

The TBCA methodology groups responses into themes to quantify them but does not allow consideration of the interaction between participants. Participants had a number of specific questions, such as the long-term outcomes of using immersive VR, the optimal target users for VR (level of disability), and the extent of translation of VR activity into “real-life” function. However, there is currently a lack of literature to provide responses to these questions, which highlights areas for future research.

### Conclusions

This is the first study exploring the views of people with MS and clinicians in terms of VR for UL rehabilitation for people with MS and has highlighted the current challenges in UL rehabilitation even though UL impairment is common and impacts meaningful activity. Overall, people with MS often found dexterity-related activities difficult, which impacted multiple ADL and challenges faced in therapy related to motivation, lack of resources, and difficulty finding interesting UL exercises. There was positive support for VR for UL exercise. Overall, to improve engagement and satisfaction for the user, this study suggests any VR games developed for people with MS should (1) be fun and engaging; (2) have clear aims related to the individual user’s goals; (3) offer personalization, such as a variety of games (abstract and ADL based), different movements, levels of difficulty, and methods of feedback; (4) monitor quality as well as quantity of movement during gameplay; (5) incorporate design features to reduce the potential for cybersickness; (6) consider if the games can incorporate education or skill development; (7) incorporate aspects of social interaction; and (8) consider including haptic properties. The findings support the need for the creation of bespoke serious games rather than using commercially available exercise games, which can discourage users with motor dysfunction [15,59]. Overall, future development of VR games for UL rehabilitation should focus on a personalized and customizable approach to encourage long-term engagement to improve meaningful outcomes for people with MS.

## Appendix

Multimedia Appendix 1 Focus group interview questions for both participant groups.

Multimedia Appendix 2 Compilation of 3 videos shown to participants during focus groups showing 3 different virtual reality systems.

Multimedia Appendix 3 Main, higher order, and raw themes from theme-based content analysis of people with multiple sclerosis focus groups, with example quotes.

Multimedia Appendix 4 Main, higher order, and raw themes from theme-based content analysis of clinician focus groups, with example quotes.

## Abbreviations

ADL: activities of daily living
HMD: head-mounted device
MS: multiple sclerosis
NHS: National Health Service
SPMS: secondary progressive multiple sclerosis
TBCA: theme-based content analysis
UL: upper limb
VR: virtual reality
