# Supplementary material for: Views of Specialist Clinicians and People With Multiple Sclerosis on Upper Limb Impairment and the Potential Role of Virtual Reality in the Rehabilitation of the Upper Limb in Multiple Sclerosis: Focus Group Study
Source: JMIR Serious Games. 2024 Apr 26;12:e51508. doi: 10.2196/51508 (PMC11087863; doi:10.2196/51508)
Supplement: Multimedia Appendix 1 [file games_v12i1e51508_app1.docx]

# Multimedia Appendix 1: Focus Group Schedules

## People with MS Focus Group Schedule

**Part 1:** Upper Limb Difficulties, Current Physiotherapy and Motivations:

Question 1: Think about your daily routine. Are there any particular activities or tasks you currently find difficult to complete? Specifically think about tasks that involve your arms and hands? (Prompt: Dressing, eating, any hobbies).

Question 2: Question 2: What do you think makes these tasks difficult? (Prompt: Such as fatigue or sensory problems?)

Question 3: Do you currently have or ever had a rehabilitation or exercise regime for your arms or hands?

- *Follow Up:* If so what did it involve? What exercises/movements did you do? How many times a week/day and how long for?
- *Follow Up:* How did you find them? (Prompts: Did you find them easy, challenging, enjoyable?)

Question 4: Are you still exercising? If Yes explain why and what kept you motivated to continue doing the exercise or regime? If No, explain why not.

- *Follow Up:* Is there anything that might motivate you to exercise?
- *Follow Up:* Does competition make you more motivated to participate in exercise or physiotherapy?
- *Follow Up:* Can you think of any other things that would keep you engaged or motivated during exercise? (Prompts: rewards, personal goals; beating personal bests)

Question 5: During exercise, do you respond to verbal encouragement, examples being someone telling you to “keep trying” or “you’re doing great”? Can you explain why?

Question 6: What are your opinions on social aspects, or doing group exercises or exercise with a friend or partner, would this help you to keep doing your exercise programme?

**Part 2:** Virtual Reality

*The participants will be shown three videos: the first will be MS participants using a Leap Motion Controller only; the second will be a video of the Leap Motion used with a virtual reality headset; then finally the last video shown will be gameplay and demonstration of the Oculus Quest virtual reality headset. Each video will last approximately 3 minutes and the context of each video, and the specific technology used, will be described before showing.*

[Show Video 1]

Question 8: What are your initial thoughts on this?

[Show Video 2]

Question 9: What are your initial thoughts on this?

[Show Video 3]

Question 10: What are your initial thoughts on this?

Question 11: After watching these videos, which virtual reality approach did you find the most appealing? (Prompt: Can you explain why?)

Question 12: What are your thoughts on virtual reality for arm/hand rehabilitation?

- *Follow Up:* Why do you feel this way?

Question 13: What benefits or advantages do you believe virtual reality can offer yourself and others with MS in rehabilitation over other traditional methods (such as general exercises)? (Prompt: novelty; enjoyable; fun; escapism?)

Question 14: What concerns do you have about using virtual reality in rehabilitation?

Question 15: What are your feelings with regards to wearing a virtual reality headset, demonstrated in the videos?

Question 16: Are there any issues you believe yourself and other people with MS could face when using this type of equipment supervised by a healthcare professional such as physiotherapist, occupational therapist or unsupervised?

Question 17: Would you personally be willing to participate in a virtual reality approach to rehabilitation, and would you be able to give an explanation why?

Question 18: What are your thoughts regarding using this approach at home?

**Part 3:** Functional Requirements and Pre-Development of Games

Question 19: For the rehabilitation games, should they be realistic real-world simulations that reflect everyday activities (*show example of kitchen scene in Leap Motion Games, Webster et al., 2019; Piano game in video 3*) or abstract game play that does not directly reflect a real world activity but still incorporates movements (*show example of bubble pop game in Leap Motion Games, Webster et al., 2019*)?

Question 20: During games, you often receive a number score to indicate how many successful actions you performed. How important would knowing your score be during gameplay?

- *Follow Up:* How do you feel about feedback being optional to the user?
- *Follow Up:* Can you think of any other elements of games that could be optional/personalised? (Prompt: different exercises, difficulty levels)

Question 21: What other feedback would be beneficial to yourselves when playing these games? (Prompt: Time taken for activity or overall game play, audio feedback)

- *Follow Up:* Should this feedback be positive scoring or record the amount of errors made?

Question 22: How important is knowing what muscle group(s) is/are being targeted in each game? Why?

Question 23: The technology we will be using to develop the games will be a headset within built hand tracking, shown in the final video that showed a piano activity and moving your hand through a maze. What hand or arm movements do you believe could work well in rehabilitation games using this specific piece of technology?

[End of Focus Group]

## Clinician Focus Group Schedule

**Part 1:** Upper Limb Movements in Rehabilitation/Physiotherapy for People with MS

Question 1: What are the most common upper limb movements that are included in an upper limb rehabilitation regime for people with MS? And what are these actions aimed to specifically improve? Such as gross movement, dexterity, bimanual coordination and muscle strength?

Question 2: What are some of the challenges you face as a physiotherapy/occupational therapist when rehabilitating people with MS? (**Prompt**: Motivating the person; patient compliance?)

Question 3: What are some of the challenges you believe people with MS face when undergoing physiotherapy/occupational therapy? (**Prompt**: Symptoms such as fatigue, or themselves keeping motivated?)

**Part 2:** Virtual Reality Opinion

*The participants will be shown three videos: the first will be MS participants using a Leap Motion Controller only; the second will be a video of the Leap Motion used with a virtual reality headset; then finally the last video shown will be gameplay and demonstration of the Oculus Quest virtual reality headset. Each video will last approximately 3 minutes and the context of each video, and the specific technology used, will be described before showing.*

[Show Video 1, 2 and 3]

Question 4: What are your initial thoughts from seeing these three different approaches to rehabilitation using virtual reality? (**Prompt**: Can you explain why?)

Question 5: Does anyone have experience with using virtual reality in an exercise programme or research? If Yes, please explain what that detailed and your experience.

Question 6: What are your thoughts on virtual reality for rehabilitation an alternative to, or as an adjunct to usual care?

- *Follow Up:* Why do you feel this way?

Question 7: What benefits or advantages do you believe virtual reality can offer in rehabilitation over other traditional methods (such as general exercises)? (**Prompt**: novelty; enjoyable; fun; escapism?)

Question 8: What concerns do you have about using virtual reality in rehabilitation? (**Prompt**: Concerns about the technology, patient issues, safety)

Question 9: What are your feelings with regards to people with MS wearing a virtual reality headset, demonstrated in the videos?

Question 10: What are your thoughts regarding using virtual reality as a home-based approach?

**Part 3:** Functional Requirements and Pre-Development of Games

Question 11: For the rehabilitation games, there could be realistic real-world simulations that reflect everyday activities (*show example of kitchen scene in Leap Motion Games, Webster et al., 2019; Piano game in video 3*) or could be abstract game play that does not directly reflect a real world activity but still incorporates movements (*show example of bubble pop game in Leap Motion Games, Webster et al., 2019*).. What barriers do you think virtual reality would have in providing real world simulations for rehabilitation and the abstract approach to rehabilitation? (**Prompt**: Frustration in translation between virtual and real world and subjectivity in interest with specific game; patients may not see the direct benefit from the task within the game?))

Question 12: What type of feedback would you suggest to implement into rehabilitation games? (**Prompt**: scoring, time, audio)

- *Follow Up:* Should this feedback be positive scoring or record the amount of errors made?

Question 13: Do you believe that such feedback should be optional for the patient? Why?

- *Follow Up:* Can you think of any other factors that could be optional/personalised? (**Prompt**: different exercises, difficulty levels).

Question 14: How important is knowing what muscle group(s) is/are being targeted in each game for the patient? Why?

Question 15: What role do you see yourselves as physiotherapists/occupational therapists having in a regime with virtual reality based games?

- *Follow Up:* What would be your feelings to having to personally use virtual reality as part of rehabilitation for MS patients?

Question 16: What information from a patient’s session using the virtual reality games would you like to see relayed back to yourself?

Question 17: What barriers do you predict yourselves and people with MS will face when using virtual reality games as part of rehabilitation?

Question 18: The technology we will be using to develop the games will be a headset within built hand tracking, shown in the final video that showed a piano activity and moving your hand through a maze. What other hand or arm movements or tasks do you believe could work well in rehabilitation games using this specific piece of technology?

[End of Focus Group]
