# Supplementary material for: Views of Specialist Clinicians and People With Multiple Sclerosis on Upper Limb Impairment and the Potential Role of Virtual Reality in the Rehabilitation of the Upper Limb in Multiple Sclerosis: Focus Group Study
Source: JMIR Serious Games. 2024 Apr 26;12:e51508. doi: 10.2196/51508 (PMC11087863; doi:10.2196/51508)
Supplement: Multimedia Appendix 3 [file games_v12i1e51508_app3.docx]

Multimedia Appendix 3**:** Main, Higher Order and Raw Themes from TBCA People with MS Focus Groups, with example quotes

| **Main Theme** | **Higher Order Themes (No. of Responses)** | **Raw Themes (No. of Responses)** | **Example Quote** |
| --- | --- | --- | --- |
| Impact of MS on the Upper Limb | Interference with Functional Activities (35) | Dressing (8); Eating (6); Dropping Items (5); Writing (5); Grooming (3); Dependence on Others for ADLs (3); Carrying Items (3); Travelling (2) | “I struggle to eat and keeping the fork and that steady because of my tremors” (P10, age 84 female with SPMS) |
|  | Symptoms and Signs that Impact Activities (25) | Fatigue (10); Numbness (6); Sensory Overload (4); Weakness (3); Tremors (2); Proprioception (2); Coordination (1) | “I have to watch what I’m doing I can maybe manage but if I try to tie a lace, while I’m doing, if I’m looking away… that’s just not possible… it’s a kind of sensory and a visual thing” (P4, age 58 female with SPMS) |
|  | Strategies People with MS Adopt to Assist with Activities of Daily Living (24) | Strategies for Functional Activities (8); Adapting (7); Making Difference (5); Technology Assistance (2); Mobility-assistance Equipment (2) | “I’ve learned to shave with my left hand, I use the computer mouse with my left hand… I’ve tried to learn how to write with my left hand but that’s not been very successful… I suppose compensating rather than… trying to retrain” (P9, age 60 male with SPMS) |
|  | Struggle with Loss of Meaningful Activities and Skills (14) | Loss of Skills (6); Impact of Losing Ability to Write (4); Keeping Meaningful Activities (4) | “I used to be a writer and it was very, very hard because I couldn’t write anymore… I was really motivated [to relearn writing], felt really cut off from the world” (P8, age 70 male with PPMS) |
|  | Upper Limb Actions People with MS Find Difficult (13) | Dexterity (6); Range of Motion (4); Grip (3) | “I just find it difficult to grasp things” (P4, age 58 female with SPMS) |
|  | Sharing and Sympathy (13) | Sharing Strategies (4); Sharing Advice on Exercise (4); Taking Advice (3); Sympathising (2) | “I do recommend those the… they’re very useful the MS Society videos” (P8, age 70 male with PPMS) |
|  | Difficulty with Progression and Unpredictable nature of MS (10) | Variation in MS (6); Unpredictable (2);  Progression (2) | “We can see today everybody’s MS is completely different… there’s no two people identical” (P3, age 68 male with SPMS) |
|  |  |  |  |
| Exercising with MS | Views and Attitudes on Exercise (49) | Maintenance (10); Negative Perceptions of exercise (8); Keeping muscle strength (8); Determined to Exercise (7); Benefits of Exercise (6); Multitask Approach (4); Legs Focus (3); In control (3) | “I’m able to motivate myself because my, my mobility in my legs has gone so quickly but when it comes to my arms I’m much less motivated to do stuff… if I stop my legs I can’t walk at all and then my world goes, whereas if it’s my hands and things like that I don’t feel as motivated to do it” (P6, age 28 female with RRMS) |
|  | Previous Experience of Upper Limb Rehabilitation/ Exercise (40) | Outcomes from UL Exercise/Rehab (12); Neglecting UL Exercise or Rehabilitation (10); UL Equipment (6); UL Physiotherapy (4); Driven for UL exercise (3); UL Exercise Resources (3); Adherence (2) | “I couldn’t hold a kettle… I couldn’t do it with my right hand before I started the [MS Society video] exercise and now I can” (P8, age 70 male with PPMS) |
|  | Barriers to Exercise (28) | Personal Barriers (8); Environmental Barriers (8); COVID Barriers (7); Verbal Disengagement (5) | “Before COVID we actually had me walking a few steps with my crutches rather than being in a wheelchair – which is a massive, massive, massive difference for me but then of course COVID hit and now I’m back to square one” (P5, age 42 female with SPMS) |
|  | Facilitators to Exercise (28) | Verbal Encouragement (10); Health Care Professionals (8); MS Centre (4); Gym Facilitators (3); Pushing self for Results (3) | “I think if you’ve got someone pushing you, sort of giving you keep going keep going. Making you go further and if nobody’s there you’re just had enough. But if somebody’s there saying oh no that does help” (P7, age 56 female with PPMS) |
|  | Adverse Effects of Exercise (11) | Induce Symptoms (4); Tiring (3); Recovery time after exercise (2); Affecting Socializing (1); Overdoing Exercise (1) | “When I was at [hospital] they almost knackered me. They, although they’re trying very hard, they don’t know me well and they overdid it” (P3, age 68 male with SPMS) |
|  | Approaches to Exercise used by People with MS (26) | Routine (7); Exercise Bikes (6); Exercise Aims (5); Low Impact/Stretching Exercise (4); Physiotherapy Approaches (4) | “I make a point of either going out with the dog after work or going, making a point of going to the gym because I have been sitting all day so” (P2, age 38 female with RRMS) |
|  | Views on Group versus Individual Exercise (26) | Competition in Exercise (10); Motivation of group exercise (5); Downsides of group Exercise (5); Importance of socializing in Exercise (2); Camaraderie (2); Enjoyment (1); Interest in Group Exercise (1) | “I’m not too fussed about being in competition with others, but if it was a more social thing that would maybe encourage me to perhaps join in a group that’s doing something together” (P4, age 58 female with SPMS) |
|  |  |  |  |
| People with MS’ Views on VR | Positive Views on VR (55) | Home Use (9); Outcome Benefits (6); Personal Opinions on VR (5); Fun (5); Adaptable (5); Positives of Technology (5); Wireless Convenience (5); Accessibility Convenience (4); Incentives (3); Meaningful (3); Online Socializing (2); Immersion (1) | “I think it’s [VR] still very good because… it’s… maintaining those motor skills that is so easily slip away when you’re not using them” (P9, age 60 male with SPMS) |
|  | Negative Views on VR (40) | Cybersickness (17); HMD Discomfort (6); Technology Discomfort (5); HMD Dislike (3); Disengagement (3); Accessibility Concerns (3); Unsuitability (3) | “I have suffered a lot of balance issues so something like that on my head and trying to do the link between something on your head and moving your arms on a screen. That might just make me a bit dizzy” (P4, age 58 female with SPMS) |
|  | Views on Trying or Participating in VR Rehabilitation (25) | Openness to VR (12); Challenging (4); Safety Considerations (3); Need Results (2); Technology Considerations (2); Unsuitable for them (2) | “I’d be interested in buying it, even if it’s just the challenge of it I’d be interested in trying” (P10, age 84 female with SPMS) |
|  |  |  |  |
| People with MS’ Recommendations for Development and User Requirements | Considerations for Development of VR Games (84) | Mindful of Target Audience (9);Tracking Progress (8); Discouragement of Feedback (8); Knowing UL Outcomes (7); End Result (6); Score Targets (6);  Challenging Self (6); Competition in Games (5); Education (5);  Time Feedback (4); Supervision (4) Community Involvement (3); Multipurpose (3); Continuous development (3); Be Fun (3); Hardware (2); Learning Patterns Concern (2) | “Age demographic for people who are diagnosed is…20 to 30. So you’ve got to kinda get in there, and kinda make something that works for them. Whereas, yeah, the older generations maybe…it’s more basic stuff” (P6, age 28 female with RRMS) |
|  | Suggestions for VR Activities (36) | Suggested UL Actions (9); Game Ideas (7); Real Life vs. Abstract Tasks (4); Haptic Activities (4); Strength in Games (4); Writing and Drawing (3); Demonstrated Games (3); Additional Objectives (2); Atmosphere (1) | “Pushing down something like the whack-a-mole sort of thing? Something like that?... Or the what’s the thing the fairground, the hit the bell thing?... You hit it and one lights up or you hit or something” (P2, age 38 female with RRMS) |
|  | Importance of Choice (23) | Offer Different Movements (8); Having Variety of Games (6); Personal preferences (6); Variety of Different Levels (3) | “I’d like to make sure I’m not doing a whole lot of exercises that are all doing the same things… Got to be mixing them up: one for coordination, one for dexterity” (P1, age 60 female with SPMS) |

Abbreviations: HMD (Head mounted device); MS (Multiple sclerosis); PPMS (Primary progressive multiple sclerosis); RRMS (Relapse and remitting multiple sclerosis); SPMS (Secondary progressive multiple sclerosis); UL (Upper Limb); VR (Virtual reality).
