# Supplementary material for: Views of Specialist Clinicians and People With Multiple Sclerosis on Upper Limb Impairment and the Potential Role of Virtual Reality in the Rehabilitation of the Upper Limb in Multiple Sclerosis: Focus Group Study
Source: JMIR Serious Games. 2024 Apr 26;12:e51508. doi: 10.2196/51508 (PMC11087863; doi:10.2196/51508)
Supplement: Multimedia Appendix 4 [file games_v12i1e51508_app4.docx]

Multimedia Appendix 4**:** Main, Higher Order and Raw Themes from TBCA Clinician Focus Groups, with example quotes

| **Main Themes** | **Higher Order Themes (No. of Responses)** | **Raw Themes (No. of Responses)** | **Example Quote** |
| --- | --- | --- | --- |
| Current Methods and Challenges for Delivering Upper Limb Rehabilitation | Challenges Clinicians Face when Delivering Exercise for People with MS (52) | MS-specific challenges (13); Patient Adherence (11); Service Challenges (9); UL related challenges (7); Patient Differences (6); Challenges with current methods of delivery (4); COVID impacts (2) | “A bit more difficult for upper limb things… it’s much easier to maybe… go for a walk with somebody or you know, or cycle or whatever. Upper limb is maybe a wee bit more difficult” (C6, PT) |
|  | Recommended Upper Limb Exercises for People with MS (29) | Actions (10); Systematic Approach (7); Functional Tasks (6); Strength and range of movement (5); Relapse care (1) | “Looking at strength, coordination, proprioception, muscle tone, muscle strength, range of movement all of those things” (C6, PT) |
|  | Experience with Long Term, Progressive condition (24) | Deterioration (11); Acceptance in Patients (8); Difficulty with Patient Improvements (5) | “There is that assumption that if they fight, work hard, push through, all that language that people with MS tend to use, that they will be the person who doesn’t end up with progressive MS. And then the realisation that doesn’t happen can be really challenging” (C5, OT) |
|  | Factors Clinicians Consider when Prescribing Exercise for the Upper Limb (22) | Meaningful and Patient Focused (9); Patient Assessments (6); Symptoms (4); Repetition (3) | “You would probably break it down quite systematically and just work on key areas of weakness” (C3, PT) |
|  | Current Methods of Upper Limb Exercise Delivery for People with MS (15) | Technological Approaches (4); Programmes (4); Accessible Equipment (3); Clinician Routines (2); Patient Lead (2) | “So [UL rehabilitation] it’s community based but very little equipment so either body weight stuff or whatever we could find to hand – water bottles or TheraBands” (C7, PT) |
|  | Socializing in Exercise (14) | Social motivation (6); Support (5); Recommending social exercise (3) | “Are able to maintain [social meetings] when one of them suddenly has a dip… the power of that, of people with the same condition, difficulties that really understand” (C5, OT) |
|  | | | |
| Clinicians’ Views on Virtual Reality | Positive Views on VR (50) | Solutions to current challenges (10); Personal Opinions on VR (7); Facilitating movements/tasks (6); VR-specific qualities (6); Meaningful (5); Engagement (5); Visualisation (4); Novel (3); Cognitive appeal (2); Adaptability (2) | “What appeals about VR stuff is that it is focused and takes you into a different place… You’re doing tai chi on a beautiful, Japanese garden rather than actually in your grumpy living room… I think even that in terms of the escapism aspect, maybe from a mental wellbeing” (C1, PT) |
|  | Negative Views on VR (38) | Disengagement (10); Cybersickness and safety (8); HMD discomfort (7); Accessibility concerns (5); Feedback concerns (5); Validity concerns (3) | “There’s some sort of earlier papers looking at VR in neurorehab… their outcomes were indicating that the benefits, the benefits people gain from doing VR simulations were very specific to that activity so some of the outcomes were not necessarily transferrable into real function” (C1, PT) |
|  | Questioning Benefits and the Unknowns of VR (14) | Questioning Purpose of VR (4); Questioning Benefits of VR (4); Neural Mechanisms (3); Research (2); Different VR systems (1) | “I think it’s important to think about how is [VR] different to just doing [activities] in real life as well… What can you augment in your rehab through this virtual reality that you can’t just do in real life anyway?” (C7, PT) |
| Clinicians’ Recommendations for Development and User Requirements | Considerations for Developing VR Games for People with MS (41) | Communication between clinician and patient (12); Purposeful (7); Social components (7); Selecting tasks (4); Slower tasks (3); Competition (3); Feedback for clinician (3); Positive Feedback (2); End point (2) | “If it’s designed to be fun, if you can either interact virtually with your therapist or other people then I’m sure that would be quite good” (C8, OT) |
|  | Suggestions for VR Activities (18) | ADL Activities (6); Hobbies (6); Objectives (6) | “Maybe, like, a supermarket… there could be reaching but then there might be fruit where you need to do more fine finger movements?” (C7, PT) |
|  | Importance of Choice (15) | Preferences (6); Having Variety (5); Set Up (4) | “I think, it is about having a variety of things that push as many buttons with patients that you can manage and cover as many options as you can” (C2, PT) |
|  |  |  |  |
| Implementation of VR into Practice | Suggestions for Incorporation of VR into Practice (18) | Home use (7); VR in Clinics (7); Long term treatment (4); | “If you were using it at home you could use this style of interview [on Microsoft Teams] where you like: right show me what you’re doing, let’s have a look at it. It’s quicker process to go through” (C3, PT) |
|  | Challenges with Implementation of VR into Practice (24) | Funding (7); Demanding on Services (6); Availability of Equipment (5); Risk (3); Adjustment (2); Uncertainty of Practice (2) | “I know if I brought it to my bosses they would want a breakdown of cost of monthly rate, how are we going to utilise it, how often are we going to utilise it. What figures could we get from this particular item and what outcomes could we achieve” (C4, MS Specialist Nurse) |
|  | Finding the Target Audience for VR (8) | Who would use VR (3); Niche Group (3); Age (2) | “I think there certainly would be people who were interested in it, but I think, who would be, who would be using it?” (C8, OT) |

Abbreviations: C (Clinician); HMD (Head mounted device); OT (Occupational therapist); PT (physiotherapist); UL (Upper limb); VR (Virtual reality).
